# 

**Published:** 1880-10

**Authors:** 


### BOOKS AND PAMPHLETS RECEIVED.

**Atlas of Human Anatomy,** (parts v to xiv.) Arranged according to Drs. OESTERREICHER and ERDL; containing 180 large plates. A. E. Wilde & Co., Cincinnati.

**Eighth Annual Report** relating to the registry and return of births, marriages and deaths in Michigan, for the year 1874, by the Superintendent of Vital Statistics. HENRY B. BAKER, M. D.

**Malaria and its Effects.** J. W. YOUNGS, M. D.

**The Invention of Anæsthetic Inhalation.** By WILLIAM T. MORTON, M. D. D. Appleton & Co., New York.

**Hygiene of Catarrh.** By THOS. F. RUMBOLD, M. D. Geo. O. Rumbold & Co., St. Louis.

**Atlas of Skin Diseases.** LOUIS A. DUHRING, M. D. Parts vii. J. B. Lippincott & Co., Philadelphia.

**Transactions Missouri State Medical Association, 1880.**

